# Development of an effective virtual environment in eliciting craving in adolescents and young adults with internet gaming disorder

**DOI:** 10.1371/journal.pone.0195677

**Published:** 2018-04-19

**Authors:** Yu-Bin Shin, Jae-Jin Kim, Min-Kyeong Kim, Sunghyon Kyeong, Young Hoon Jung, Hyojung Eom, Eunjoo Kim

**Affiliations:** 1 Brain Korea 21 PLUS Project for Medical Science, Yonsei University, Seoul, Republic of Korea; 2 Institute of Behavioral Science in Medicine, Yonsei University College of Medicine, Seoul, Republic of Korea; 3 Department of Psychiatry, Yonsei University Gangnam Severance Hospital, Seoul, Republic of Korea; Centre for Addiction and Mental Health, CANADA

## Abstract

Internet gaming disorder (IGD) is a new disorder that warrants further investigation, as recently noted in the research criteria of the Diagnostic and Statistical Manual of Mental Disorders, Fifth Edition. Offering controlled environments that increase cue-induced craving, virtual reality cue-exposure therapy has been shown to be effective for some addiction disorders. To assess the feasibility of virtual reality for patients with IGD, this study aimed to develop virtual environments that represent risk situations for inducing craving, and assess the effect of virtual reality in cue reactivity. A total of 64 male adolescents and young adults (34 with IGD and 30 without) were recruited for participation. We developed a virtual internet café environment and the participants were exposed to four different tasks. As the primary feasibility outcome, cravings were measured with a visual analogue scale measuring current urge to play a game after exposure to each task. The virtual internet café induced significantly greater cravings in patients with IGD compared to controls. Additionally, patients exhibited a significantly higher acceptance rate of an avatar’s invitation to play a game together than that of controls. In IGD, craving response to the tasks was positively associated with the symptom severity score as measured by Young's Internet Addiction Test. These findings reveal that virtual reality laden with complex game-related cues could evoke game craving in patients with IGD and could be used in the treatment of IGD as a cue-exposure therapy tool for eliciting craving.

## Introduction

Internet gaming disorder (IGD), as recently included in the appendix of the Diagnostic and Statistical Manual of Mental Disorders, Fifth Edition (DSM-5), has become a serious public health problem [1–3]. Because of a rapid rise in internet use, especially among adolescents and young adults, maladaptive use of the internet most commonly occurs in this developmental period [4]. As a transitional developmental stage between childhood and adulthood, adolescence is a critical period for addiction vulnerability [5]. Several studies have reported that adolescents and young adults generally show higher rates of experimentation with and problematic use of substances and gambling than older adults, and adults with a diagnosis of addictive disorders commonly show onset during this period [6,7]. As social, behavioral, and neurobiological development continues throughout adolescence and young adulthood, an addictive disorder during this time could even lead to alteration in brain organization and function [8,9]. Therefore, research and treatment targeting adolescents and young adults with addictive disorders may provide life-long benefits.

There has been some debate as to whether IGD should be included in the category of addictive disorders. According to a recent review, behavioral tendency toward game-related cues and cue-induced neurobiological changes in internet gaming abusers are similar to those seen in people with substance-use disorder (SUD) and pathological gambling (PG), suggesting that IGD may share behavioral and neural mechanisms with SUD and PG [10]. Moreover, internet gaming also stimulates the dopamine-mediated mesolimbic trajectory associated with drug addiction [11,12]. Therefore, studies of IGD-targeted treatment mechanisms are needed to help with the classification of behavioral addiction and the recovery of patients with IGD. In this context, substantial effort has been devoted to developing treatments and identifying predictors of relapse in addiction research [13,14]. It has been reported that one of the strongest predictors of relapse is craving. Craving, defined as a strong desire or urge to use a specific substance or gamble, is a new criterion that was added to DSM-5 for the diagnosis of SUD and PG [1]. Thus, many of the effective treatments for addiction have focused on reducing and managing craving.

Designed to reduce craving, cue-exposure therapy involves controlled and repeated exposure to cues relating to addiction [15]. The cue-reactivity paradigm has mostly focused on proximal cues that are directly linked to actual addictive behaviors (e.g., in the case of a cigarette smoker, burning cigarettes, ashtrays, lighters, or smoking behavior) because such cues are presumably ubiquitous throughout all addictive experience [16,17]. However, craving can be provoked by other cues. Distal cues can be defined as stimuli that have been regularly present during substance use, such as a physical environment, situations, or feelings [18]. Thus, as an alternative to traditional exposure techniques using in vivo, visual, or imaginal modes of cue presentation, recent research has highlighted the usefulness of virtual reality techniques for generating more realistic craving states by providing complex cues [19].

Several investigations have demonstrated the effectiveness of virtual reality (VR) in increasing craving reactivity [20,21]. For example, the efficacy of a virtual environment (VE) reproducing high-risk situations (e.g., being in a virtual pub where diverse forms of smoking paraphernalia and avatars who are offering cigarettes are displayed) has been documented for eliciting subjective craving in patients with SUD [22] and PG [23]. Moreover, studies have demonstrated the efficacy of virtual reality cue-exposure therapy (VRCET) for SUD and PG in reducing craving in response to addiction-related cues and extinction of cue-response association [24,25]. Furthermore, VRCET was found to be as effective as conventional therapy in the treatment of nicotine dependence [26], and CBT coupled with VRCET is effective in the prevention of smoking relapse [27]. Thus, the cue-exposure technique is now being applied to the other clinical targets, such as the IGD [10]. For example, behavioral interventions involving craving induction [28] and virtual reality therapy [29] have been shown to be efficacious, not only in decreasing craving and severity of internet addiction, but also in altering neural activity and functional connectivity. However, few studies have been conducted to specifically investigate use of a VE to elicit cue-induced gaming craving in adolescents and young adults with IGD.

Regarding the location of internet use, most young people use the internet at home, but they also go to internet cafes where they can freely use the internet without parental monitoring [30–34]. Recent studies have demonstrated that access via the internet café is a significant predictor for high internet users [31]. Moreover, as peer influence is one of the factors most commonly linked to risky behavior in adolescents and young adults [35], the internet café environment could enable young people to satisfy a need for acceptance or social support while playing games with peers [33]. Therefore, the urge to game may arise from exposure to a virtual internet cafe that presents gaming-related events commonly experienced by young people. Given that VR could provide patients with opportunities to safely develop and practice coping skills while immersed in an ecologically valid environment [36,37], identifying features of a valid and reliable VE, as a medium for game craving assessment and graded exposure therapy for craving reduction, is essential for future studies.

In addition, there have been relatively few studies of the relationship between cue reactivity and symptom severity [38,39]. A recent study has shown that severity of disorder predicts craving in a VE for cigarette-deprived smokers [40] and gamblers [41]. These studies suggest that investigation of the relationship between IGD severity and cue-reactivity is important to generalize these results in the literature on VRCET.

Therefore, this study explored the feasibility of VR for adolescents and young adults with IGD. For this purpose, we aimed to examine whether adolescents and young adults with IGD react to a virtual internet cafe differently compared with controls. In addition, as preliminary data on the potential of a virtual internet café as a therapeutic tool, brief practice session using a commonly used coping strategy (e.g. refusal skill training in the treatment of addictive disorder) was implemented in our VR program, and we evaluated whether participants’ response could be changed after this practice. A second aim was to assess the extent to which self-reported measures of severity of internet addiction are correlated with VR-induced craving.

## Methods

### Ethics statement

The study was approved by the Institutional Review Board of Yonsei University Gangnam Severance Hospital, and written informed consent was obtained from all participants after they had received a detailed explanation of the study. In case of adolescents, the written informed consent of their parents was also obtained.

### Participants

Male adolescents and young adults (12–25 years of age) were recruited through advertisement at a local hospital and via the internet. As males have a higher prevalence of problematic use of internet games compared with females, only male participants were recruited [42]. All participants were interviewed by a psychiatrist for IGD diagnosis according to DSM-5 section 3 [1]. All enrolled IGD participants were currently addicted to one of Korea’s popular internet games (e.g., League of Legends, Overwatch, or Sudden Attack, etc.) and smartphone games (e.g., Modoo Marble, Clash of Clans, or Seven Knights, etc.). For controls, they were recruited from the local community using online advertisements and psychiatric interviews, and reviews of history were performed to confirm that none had ever been diagnosed with IGD. In addition, controls were screened for family history of psychiatric disorders, and no one had history of any psychiatric illness in first-degree relatives. Exclusion criteria for controls were current use of psychotropic medication and any history of substance use disorder, neurological or neurodevelopmental disorder, major depressive episodes, bipolar I disorder, and psychotic disorder, according to the Mini-International Neuropsychiatric Interview for Children and Adolescents (MINI-KID 6.0) for participants under the age of 18 and the Mini-International Neuropsychiatric Interview (MINI 5.0) for adult participants [43,44]. However, IGD could be highly comorbid with other mental disorders, with the DSM-5, specifically mentioning major depressive disorder and attention deficit hyperactivity disorder. Therefore, in this study, some participants show comorbidity of other mental disorders, as seen in Table 1. Participant characteristics by group are reported in Table 1.

**Table 1:** Demographic and clinical characteristics, and virtual reality experience assessments of IGD versus HC.

		IGD (n = 34)	HC (n = 30)	χ^2^	p
		N (%)	N (%)		
**Age group**				.22	.63
	**Adolescents**	19 (44.11)	15 (50.00)		
	**Young adults**	15 (55.88)	15 (50.00)		
**Any comorbidity**				14.39	<.001
	**MDD**	9 (26.47)	0		
	**ADHD**	4 (11.76)	0		
		**Mean (SD)**	**Mean (SD)**	***t***	***p***
**Age (years)**		17.20 (4.57)	18.60 (4.98)	1.16	.24
**Intelligence quotient**		111.06 (13.03)	114.13 (10.35)	1.02	.30
**Years of education**		10.97 (3.71)	11.60 (3.89)	.66	.51
**Number of hours gaming per week**		27.85 (15.85)	5.17 (6.12)	-7.33	<.001
**Internet addiction test**		55.51 (15.03)	35.07 (11.5)	-5.73	<.001
**IAT__gaming_**		59.02 (14.58)	31.86 (13.16)	-7.77	<.001
**Presence questionnaire**		135.06 (21.56)	135.96 (16.67)	.18	.85
**Simulator sickness questionnaire**		13.36 (9.70)	5.53 (6.45)	-3.73	<.001

IGD, internet gaming disorder; HC, healthy controls; SD, standard deviation; MDD, major depressive disorder; ADHD, attention-deficit/hyperactivity disorder; IAT__gaming_, modified version of internet addiction test.

### Measurements

Estimated full intellectual functioning (IQ) was obtained using the short form of the Wechsler Intelligence Scale for Children-Third Edition [45] and the Wechsler Adult Intelligence Scale-Revised [46]. Symptom severity was assessed by the Young’s Internet Addiction Test (IAT), one of the most utilized diagnostic instruments to assess the extent of internet addiction [47]. The IAT scores for 4 controls (13%) were not available because of missing data. Additionally, all participants completed a modified version of the IAT that replaced the term “Internet” in the original version with terms such as “playing internet games.” The IAT has been modified in some recent studies to assess specific subtypes of internet addiction, such as internet role-playing games (e.g., World of Warcraft) [48–50] or internet sex [51]. Cue-induced craving was assessed through a visual analogue scale (VAS) embedded into the VR. When the VAS appeared, participants were instructed to rate their urge to play game at that moment, from 0 (no urge at all) to 100 (intense urge). Participants also completed VR experience measures: the Presence Questionnaire (PQ) [52] and the Simulator Sickness Questionnaire (SSQ) [53]. To be fully immersed in a given virtual environment, it is necessary to have VR with higher sense of realism and minimal simulation sickness. Therefore, PQ and SSQ were included to assess the sense of presence and VR side effects, including some visual fatigue or motion sickness symptoms, when exposed to virtual reality.

### Virtual reality

**Fig 1:**
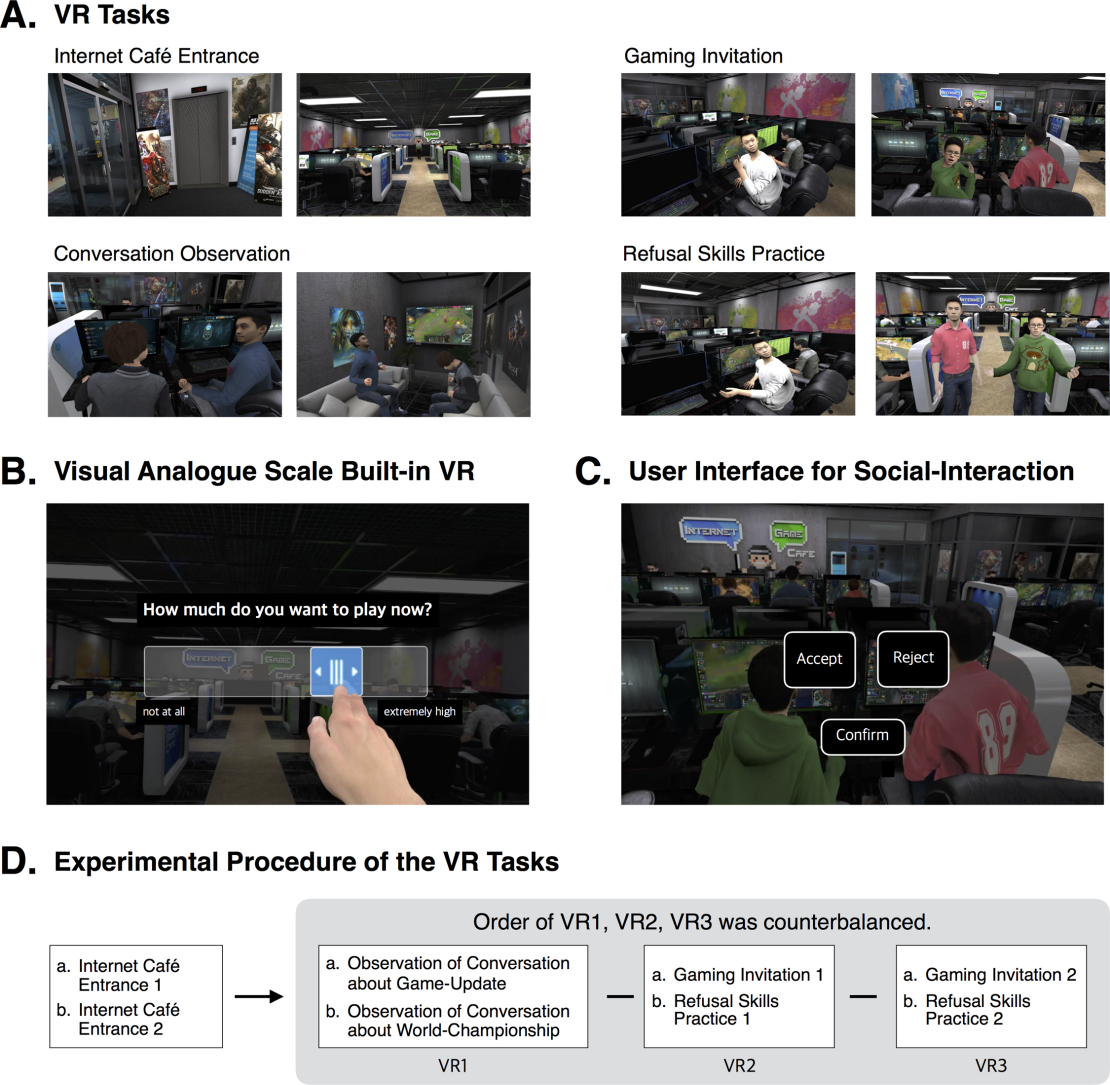
Screenshots of virtual environment and experimental procedure of virtual reality tasks.

Participants were guided through a presentation of four different sets of VR tasks in a virtual internet café (Fig 1). All participants began by finding themselves standing on the stairs of the building. The participants followed a preset pathway, climbing down the stairs and into the internet café, where the counter clerk greeted them and encouraged them to look around the internet café. In this internet café entrance task, craving was measured twice, before and after entrance. In conversation observation tasks, participants observed peers talking about game updates or world championship finals, and craving was also measured after each task.

In one of the gaming invitation tasks, the participants faced an event in which a peer pressured them to play a game together to relieve exam stress, and they were instructed to accept or reject the invitation. If they accepted the suggestion or rejected all three varying suggestions, the script ended and the VAS appeared. In the other gaming invitation task, the participants faced an event in which a peer pressured them to play a game together to win a battle against opponents who made negative comments about their game. Both tasks were identical in construct although they differed in content.

In the refusal skills practice tasks, participants were instructed to refuse avatars’ invitations to play a game together in a role-playing situation. If they refused all three varying invitations, the script ended and the VAS appeared. Both refusal skills practice tasks were identical in structure, and their content was developed based on the gaming invitation tasks. The critical difference between the gaming invitation tasks and the refusal skills practice tasks was that only in the latter were participants instructed to employ their refusal skills as a strategy for coping with the gaming invitation. This very simple intervention was delivered as a single act.

The internet café scenario was developed based on feedback from patients with IGD and clinical professionals. Especially, the virtual internet cafe generally contained game-related sounds and several avatars engaged in playing games, talking about games and surfing the internet, while the video clips shown on avatar’s monitors and TV, and the script used in conversations for the VR tasks were specifically selected from the League of Legends, the most frequently played game in Korea.

The VR system consisted of a desktop computer running the Microsoft Windows 10 operating system, containing an NVIDIA GeForce GTX 970 graphics card and 16 GB RAM of graphics memory, and an Oculus Rift head-mounted display with tracker (Oculus VR, LLC, USA). Mounted on the headset, the Leap Motion Controller (Leap Motion Inc., USA), a new device suitable for hand gesture controlled user interfaces, was used for the interactions with executable objects and avatars during the virtual experience.

### Procedure

After each participant was informed of the purpose of the study and consented to participate, the assessment of demographic and clinical characteristics and IQ was administered and participants began the VR session. As shown in Fig 1, the order in which the three sets of tasks, with the exception of the internet café entrance task, were presented was counterbalanced among participants to control order effects. Unlike the other tasks, each of the game invitation tasks was paired with one of the refusal skills practice tasks. After each VR task, self-report of craving was collected with a VAS built into the VR. After being in the VE, participants completed measures of the VR experience.

### Analysis

Differences between groups on demographic, clinical, and VR-related variables were tested using independent t-tests. To investigate the feasibility of VR for adolescents and young adults with IGD, as the primary purpose of this study, a 2 (Group: with and without IGD) × 3 (VR Task: internet café entrance, conversation observation and gaming invitation) analysis of variance (ANOVA) with repeated measures was conducted to compare the differences in self-rating of craving on the VAS as the dependent variable. Independent t-tests were used to compare differences between acceptance rates for IGD group versus controls. Effects on VR-induced cravings of strategies for coping with gaming invitations were tested using a 2 (Group: with and without IGD) × 2 (Refusal Skills: use and non-use) repeated measures ANOVA. The Refusal Skills factor reflected a comparison of craving on two different task types: gaming invitation (non-use) and refusal skills practice (use). To follow up significant main effects and interactions, post hoc analyses were conducted. Pearson correlation coefficients were used to investigate associations between dependent measures of VR-induced craving with clinical variables that differed between groups. In all cases, p<.05 (two-tailed) was considered statistically significant. For post hoc analyses, the Bonferroni correction was applied. All analyses were conducted using SPSS 23.0 (SPSS Inc., Chicago, IL).

### Results

#### Cue effects on VAS craving in a virtual internet café

A mixed-design ANOVA of VAS craving scores revealed a main effect of Group (F[1,62]=36.096, p<.001), with the IGD group having higher craving scores than controls; there was also a main effect of VR Task (F[2,124]=4.984, p=.008) and a significant Group by VR Task interaction (F[2,124]=3.263, p=.042). Post hoc analyses showed that, regardless of the group factor, entering the internet cafe (mean difference=5.031, SE=1.96, p=.035) and being invited to game (mean difference=5.678, SE=1.969, p=.014) produced significantly higher cravings than observing a conversation about internet games. In addition, among the IGD group, significantly higher cravings were reported when being invited to game compared to when observing a conversation about internet games (T[33]=-3.136, p=.004), but the VR Task differences among the control group were not significant (all *p*>.008; Table 2).

**Table 2:** Effects of virtual reality tasks on visual analogue scale (VAS) craving and acceptance rate.

		IGD (n = 34)	HC (n = 30)	t	*p*
		Mean (SD)	Mean (SD)		
**VAS Craving**					
	**Internet Café Entrance**	52.30 (24.87)	19.68 (20.48)	-5.68	<.001
	**Conversation Observation**	44.64 (26.60)	17.28 (18.93)	-4.68	<.001
	**Gaming Invitation**	55.35 (26.29)	17.93 (20.96)	-6.23	<.001
	**Refusal Skills Practice**	35.36 (28.37)	19.13 (20.72)	-2.58	.012
**Acceptance Rate (%)**		65.32 (38.30)	25.61 (33.55)	-4.38	<.001

#### Group differences in gaming invitation acceptance rates

With respect to acceptance of avatars’ invitation to play game, the IGD group showed a higher acceptance rate than controls (T[62] = -4.383, *p*<.001).

#### Effects of the refusal skills practice on VAS craving

A mixed-design ANOVA of VAS craving scores revealed a main effect of Group (F[1,62] = 22.013, p<.001) and of Refusal Skills (F[1,62]=17.456, p<.001), and a significant Group by Refusal Skills interaction (F[1,62]=22.201, p<.001). Post hoc analyses showed that craving responses to the refusal skills practice task were significantly lower than those to the gaming invitation task across both groups (mean difference=9.393, SE=2.248, p<.001) and within the IGD group (*p*<.025). In controls, no significant difference in VAS craving was found (p=.527).

#### Relationships with clinical variable

**Fig 2:**
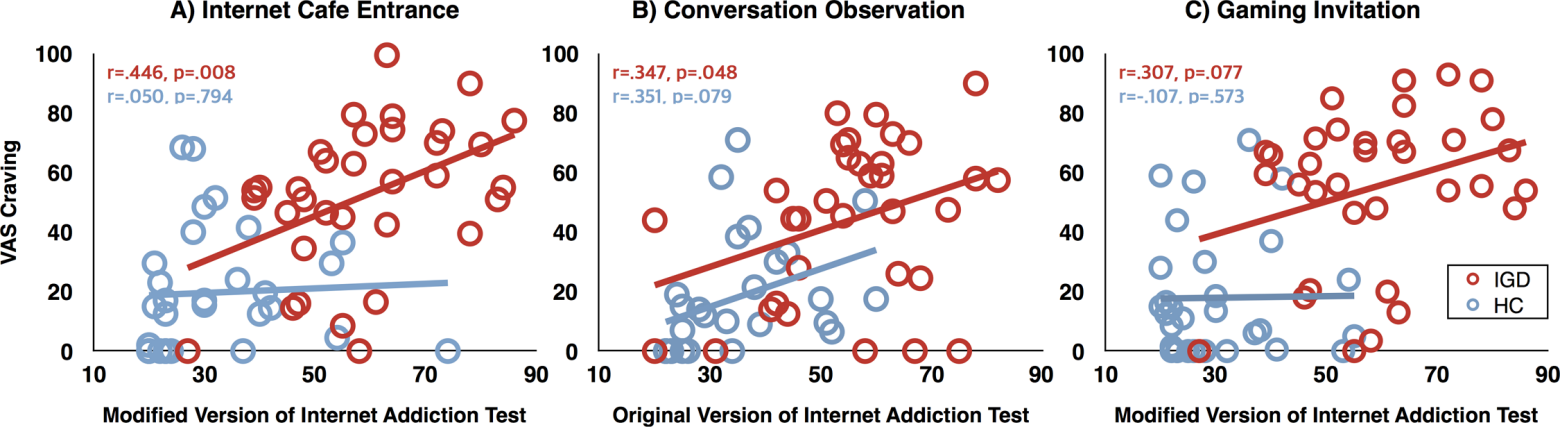
Correlations of symptom severity with visual analogue scale (VAS) craving in virtual reality.

As shown in Fig 2, within the IGD group, VAS cravings after entering the internet café were significantly positively correlated with symptom severity measured by the modified IAT (r = .446, *p* = .008), but the association of VAS cravings after gaming invitation with the modified IAT scores only reached a trend (r = .307, *p* = .077). In addition, craving to the conversation observation task was not associated with the modified IAT (r = .272, p = .120). However, VAS craving after this task was only positively associated with scores on the original version of the IAT (r = .347, p = .048) (Fig 2B). There was no relationship between VAS craving after entering the internet café or after being invited to play a game and IAT scores (all ps>.05). In controls, there were no significant correlations (all ps*>*.05).

#### Presence and simulator sickness in the VE

Participants’ average score on the PQ was 135.06 (SD = 21.56) in the IGD group and 135.96 (SD = 16.67) in controls, both with a mean score above the mid‐point of the scale. These results suggest that participants experience presence in a given virtual environment. The average score on the SSQ was 13.36 (SD = 9.70) in the IGD group and 5.53 (SD = 6.45) in controls. Scores did not differ between groups on the PQ (T[61] = .18, p = 85) but this was not the case on the SSQ (T[61] = -3.73, p<.001). Although a group difference was found on the SSQ, participants across groups reported lower levels of simulator sickness relative to established norms on the SSQ [52].

## Discussion

The primary aim of the study was to determine whether a virtual internet café was capable of eliciting cue-induced craving, and to compare these responses in individuals with IGD and healthy controls. The authors developed a VE in which game-related events that commonly experienced by adolescents and young adults were presented. The results indicated that the virtual internet café induced significantly greater craving in the IGD group than in controls. Moreover, VR-induced craving was positively associated with severity of IGD. These results suggest that an internet café scenario is useful for the evaluation of craving and document the potential of VR as a clinical tool for managing game craving.

In this study, participants were exposed to an internet café setting, and patients with IGD reported higher cravings and exhibited higher acceptance rates to gaming invitations than controls. In line with the previous studies attempting to identify the high-risk situations specific to a target disorder, these data suggest that a virtual internet café could be regarded as a high-risk situation for pathological gamers. Given the significant differences between the two groups and acceptable levels of presence and simulator sickness, the virtual internet café appears to be feasible for adolescents and young adults with IGD. Exposure to various tasks while immersed in a specific environment is important for patients to increase the treatment efficacy [25]. In this view, our results demonstrate that presentation of several addiction-related tasks relevant to the VE is also capable of inducing craving in patients with IGD.

Regardless of group, the internet café entrance task and gaming invitation task induced greater craving than the conversation observation task, which instructs participants to watch avatars talking about internet games. In the internet café entrance task, the participant follows a preset pathway, climbing down the stairs and into the internet café, which makes the virtual experience more realistic. And these VR techniques allow users to perceive that they are moving through the environment [54]. This sense of spatial presence contributes to the feeling that they are physically present in the VE and ultimately leads to a more immersive experience which makes the virtual experience more realistic. In the gaming invitation task, the participants have the opportunity to respond to the avatars’ suggestions, and this response has an immediate impact on the subsequent script (i.e., the script either ends or continues with further suggestions). These findings are consistent with the previous reports that more immersive and interactive environments contribute to large increases in the ecological validity of the VE and ultimately lead to greater craving induction [21,55]. Therefore, implementing real-time social interactions in an immersive VE may facilitate greater cue-reactivity compared to allowing the participant to perform a limited role as an observer.

Regarding the results in the IGD group, they exhibited a significant increase in craving response to the gaming invitation task compared to the conversation observation task. It has been reported that the characteristics such as substance availability, peers’ substance use, and peer group pressure, function as risk factors for addiction [6–7,35]. Given that proximal cues have strongest effects in craving induction, passive viewing of avatars who are talking about game updates and watching a gaming competition on-screen seems to be distal to IGD-related behavior. Supporting this interpretation, craving response in this task was positively associated with severity of internet addiction, not with internet gaming addiction specifically. Observing a conversation about internet games seems to reflect simulation of generic internet-related behavior. Therefore, prior to the investigation of effectiveness of VRCET using complex cues for treatment, it is important to validate the VE to make cues more salient and increase target reactivity.

In this study, patients with more severe IGD reported greater craving in the internet café entrance task, while craving in the gaming invitation task was not associated with the severity of IGD. These results may be due to an order effect, since the internet café entrance task always preceded the other tasks. Generally, initial stimuli would produce confounding effects such as familiarization, learning, or desensitization, and result in contamination of the responses of subsequent stimuli [56]. Alternatively, failure to demonstrate a relationship between the craving in the task and the symptom severity may be attributed to modified craving due to consecutive cue-exposure. This interpretation draws from the previous finding that the urge to smoke could be reduced during each of a series of virtual cue-exposure sessions [24]. Moreover, this result could be also explained by individual differences in gaming motives. Specifically, player motives are varied and some participants could be more motivated by factors other than social ones, such as rewards resulting from violent games or escapism [57]. Therefore, the order of cue presentation and the hierarchy of cues should be considered to enhance the efficacy of VRCET. With this in mind, the craving reactivity to VR gaming cues could serve as a predictive and objective measure to characterize the severity of the disorder.

Craving was significantly reduced after the refusal skills practice task; additionally, the IGD group reported greater craving across the tasks compared to controls. Moreover, although controls did not show a significant reduction in craving because of a floor effect, use of strategies for coping with invitations to game decreased craving in IGD. Examining the effects of coping strategies on VAS craving might offer additional insight into the functioning of our VR environments in IGD. It is important for people with IGD to learn refusal skills that enable them to resist peer pressure to engage in risky behaviors. Many prevention strategies focus on teaching refusal skills training in which individuals role-play turning down offers [58]. A study of smoking cessation treatment has shown VRCET with coping skills training has the potential to reduce smoking rates and craving for nicotine. Consistent with these results, our findings suggest that a virtual internet cafe could be an effective medium for the coping skills training combined with cue-exposure.

Regarding simulator sickness, SSQ evaluated several factors relating to the physical after-effects of VE exposure. It has been reported that although the SSQ measures the physical discomfort, the distressed state derived from increased craving could also be reflected in the SSQ [23,59]. In line with these studies, the higher scores reported by the IGD group could reflect some discomfort caused by elicited cravings to game.

There are some potential limitations of the current study that warrant future research. First, the differences between cue and neutral environment were not investigated. Thus, the lack of a control condition makes it difficult to generalize the present results into the literature of treatment for IGD. Second, whereas participants in this study were addicted to various kinds of internet games, the game-related stimuli implemented in the VE consisted of video clips from the League of Legends game. We did find a significant difference between the groups, regardless of specific game stimuli used for the VE. However, given that the participant’s own brand of cigarette is commonly used to make smoking cues more salient in a cue reactivity paradigm, VR using personalized game stimuli may make the VE more ecologically valid and induce more craving. Third, although it has been reported that access to the internet via internet cafés is associated with problematic internet use including internet gaming among Chinese [30], Greek [31], Taiwanese [33], and Korean [34] adolescents, the negative associations of pathological gaming with internet cafés seems to be more prevalent in Asian adolescents. Lastly, since we did not assess the inter-rater reliability during diagnosis of IGD, these results should be interpreted with caution. In order to enhance validity of IGD diagnosis, it would be helpful for multiple clinicians to diagnose IGD in future studies.

## Conclusions

In the present study, our results have demonstrated the effectiveness of a VE in eliciting cue-induced craving to game. These findings may have implications for VRCET to systematically desensitize patients with IGD. Given the feasibility demonstrated in this study of VE for IGD, future research should focus on developing VR treatments and comparing them with conventional treatment protocols.

## Supporting information

S1 Dataset The file summarizes data obtained during virtual reality cue-exposure session. (XLSX)
